# Perceived Color Gamut in Images: From Boundary to Difference

**DOI:** 10.3389/fnins.2022.907697

**Published:** 2022-06-07

**Authors:** Hao Xie, Robert Wanat, Mark D. Fairchild

**Affiliations:** ^1^Program of Color Science, Munsell Color Science Laboratory, Rochester Institute of Technology, Rochester, NY, United States; ^2^LG Electronics/Zenith R&D, San Jose, CA, United States

**Keywords:** color gamut volume, image quality, gamut boundary, image color difference, display primaries

## Abstract

A larger display color gamut volume (CGV) is expected to produce higher perceived brightness and colorfulness of the images displayed. However, display control algorithms such as gamut mapping and color conversion need to be carefully controlled to fully take advantage of the higher luminance and more saturated display primaries. Using RGBW displays (RGB plus a white channel) as a special case in contrast to RGB displays, it is demonstrated that a larger RGB display gamut enclosed by the boundary did not guarantee a larger color gamut perceived in images. Five gamuts with different white channel contributions were simulated, and seven different image contents were curated and rendered on each display. Using a paired comparison experiment with 33 observers, the perceived scales of color gamut as perceived brightness and colorfulness were derived. The results show more correlation with the image-wise than display-wise CGV and can be explained with image color differences. Our findings highlight the importance of considering image contents when optimizing display gamut volume, which can be guided by such image-wise analysis.

## 1. Introduction

Quantifying the set of colors that is reproducible on a display, i.e., the display gamut, has been useful when choosing display primaries to optimize color-related performance. Previous work mostly focused on describing the boundaries of such set, represented as either a triangle for three-primary displays on a 2D chromaticity diagram (Xie et al., 2017) or more preferably a color gamut volume (CGV) in a 3D color space (Masaoka et al., 2020). A larger CGV is expected to bring more brightness and colorfulness to the delivered images. For high dynamic range (HDR) imaging, the increase in absolute peak luminance is another factor to consider for CGV calculation.

However, when determining the final visual experience that the display offers, the resulting quality will also depend on the utilization of its rendering capability to accommodate various types of visual content, similar to how a painter chooses colors from the palette to the canvas. For example, when an image spans only a small range of colorimetric values within the gamut, further expansion of the gamut would offer little benefit to colorimetric reproduction accuracy. In general, for each image, there exist multiple ways in which any pixel color can be assigned to a color within the display gamut. Gamut mapping, color conversion, and other related operations are also critical when quantifying the perceived color gamut of images. Adopting the image quality circle concept proposed by Engeldrum (2004), the display primaries and the conversion algorithm can be considered as the physical parameters and technology variables (with a loosely defined distinction) that together impact the CGV as well as the perceptual image quality. RGBW displays, which utilize an extra white (W) channel for potential benefits in power consumption and observer metamerism, have been an interesting topic for considering display primaries and color conversion at the same time (Miller and Murdoch, 2009; Kwon et al., 2015).

In addition to gamut mapping processing, another issue regarding the relation between CGV optimization and image content is the coverage of color statistics in natural objects, such as the Pointer gamut (Pointer, 1980). It has been shown that natural image statistics correlate with perceptual sensitivity and preference (McDermott and Webster, 2012; Nascimento et al., 2021). Using natural image datasets, the distribution of dynamic range has been characterized (Grimaldi et al., 2019), however, the generalization to the chromatic dimension has not been carried out. Moreover, color reproduction does not only concern natural objects, as synthetic scenes generated by computer graphics may also benefit from a larger CGV, although the perceptual gain is not easily quantifiable.

There have been few studies considering gamut volume design and content dependency simultaneously. In this work, the relationship between computational and psychophysical quantification of the perceived gamut is examined. The importance of image content dependency is highlighted from the results. The next section describes the experiment conducted to investigate different parameters in five simulated RGBW displays and the corresponding color gamut perceived in images with 33 observers. Then, both the computational and psychophysical results, as well as the correlations are presented. The last two sections discuss and conclude our findings and the implications of considering image contents in display gamut optimization.

## 2. Gamut Calculation and Perceptual Evaluation

### 2.1. Simulated Gamut Settings

A set of five simulated display gamuts were included, including one RGB and four RGBW displays which all shared the same DCI-P3 RGB primaries. The white channel in the RGBW display was CIE D65 at varying levels of luminance, with the same total luminance of all RGB or RGBW channels as ~1, 450 nits, which corresponded to different ratios of white light output over color light output (WLO/CLO) and led to different volume sizes (Masaoka et al., 2020). WLO corresponds to the peak luminance of the display where the white channel is activated and CLO equals to the luminance of the RGB channels without the white channel contribution. A higher WLO/CLO ratio results in de-saturating the RGB primaries, thus leading to a smaller CGV. For each RGBW gamut tested, a RGB to RGBW color conversion algorithm was generated to simulate a similar conversion happening in real-world devices and to incorporate gamut mapping. The algorithm inputs were gamma-encoded RGB values and the outputs were linear RGBW, which given the RGBW primaries could be used to derive colorimetric values such as CIEXYZ for the input color. One critical parameter of the algorithm was the gamma-encoded threshold RGB value above which the white channel was being activated, mirroring a similar solution commonly used in RGBW displays. The gamut mapping primarily focused on maintaining the hue and compressing the CIELAB lightness and chroma if needed.

**Figure 1:**
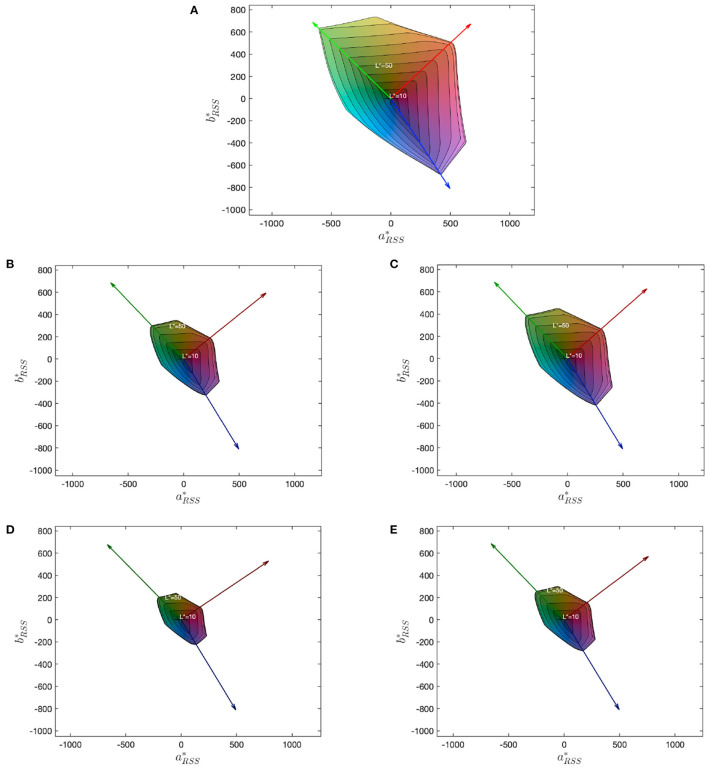
Gamut rings (Smith et al., 2020) for each gamut (**A–E** for gamut #1–5, respectively). The area of the outermost boundary in the gamut rings correspond to the CGV. The coordinates are on the CIELAB a^*^ − b^*^ plane, where the subscript *RSS* means the root sum square of the equivalent a^*^/b^*^ values (Masaoka et al., 2020). The differences between consecutive equal-lightness contours depends on both the WLO/CLO ratio and the white channel activation position.

Both the WLO/CLO variations and the activation threshold parameter resulted in different shapes of calculated CGVs in CIELAB color space despite all gamuts having the same range of luminance and primaries. Table 1 lists the parameters and the corresponding gamut volume sizes for the five simulated gamuts. Note that the gamut #2 and #3 had identical WLO/CLO ratio and similar CGVs of the enclosed volume with display boundary colors, but because they had different settings for the white channel activation threshold, they had different gamut slices at different lightness levels, which can be observed in their gamut ring visualizations in Figure 1. For each display the gamut rings were calculated which convert the 3D volume into a 2D area summation. Each ring contour corresponds to the cumulative gamut volume summed to the given lightness level and the outermost ring (L^*^ of 100 that used the peak luminance D65 as the white point) corresponds to the gamut boundary. Gamut #1, which does not use a white channel, had the largest predicted gamut volume. The difference between gamut #2 and #3 and between gamut #4 and #5 caused by the white channel activation level highlights the fact that the boundary descriptor summarized by its volume size may not capture the internal geometry of the gamut.

**Table 1:** Gamut parameter settings and the corresponding CIELAB gamut volume.

**Simulated gamut**	**#1**	**#2**	**#3**	**#4**	**#5**
WLO/CLO	1	1.5	1.5	2	2
White activation level	None	Low	High	Low	High
CGV (*10^6^)	3.72	1.23	1.76	0.75	1.01

### 2.2. Psychophysical Experiment

**Figure 2:**
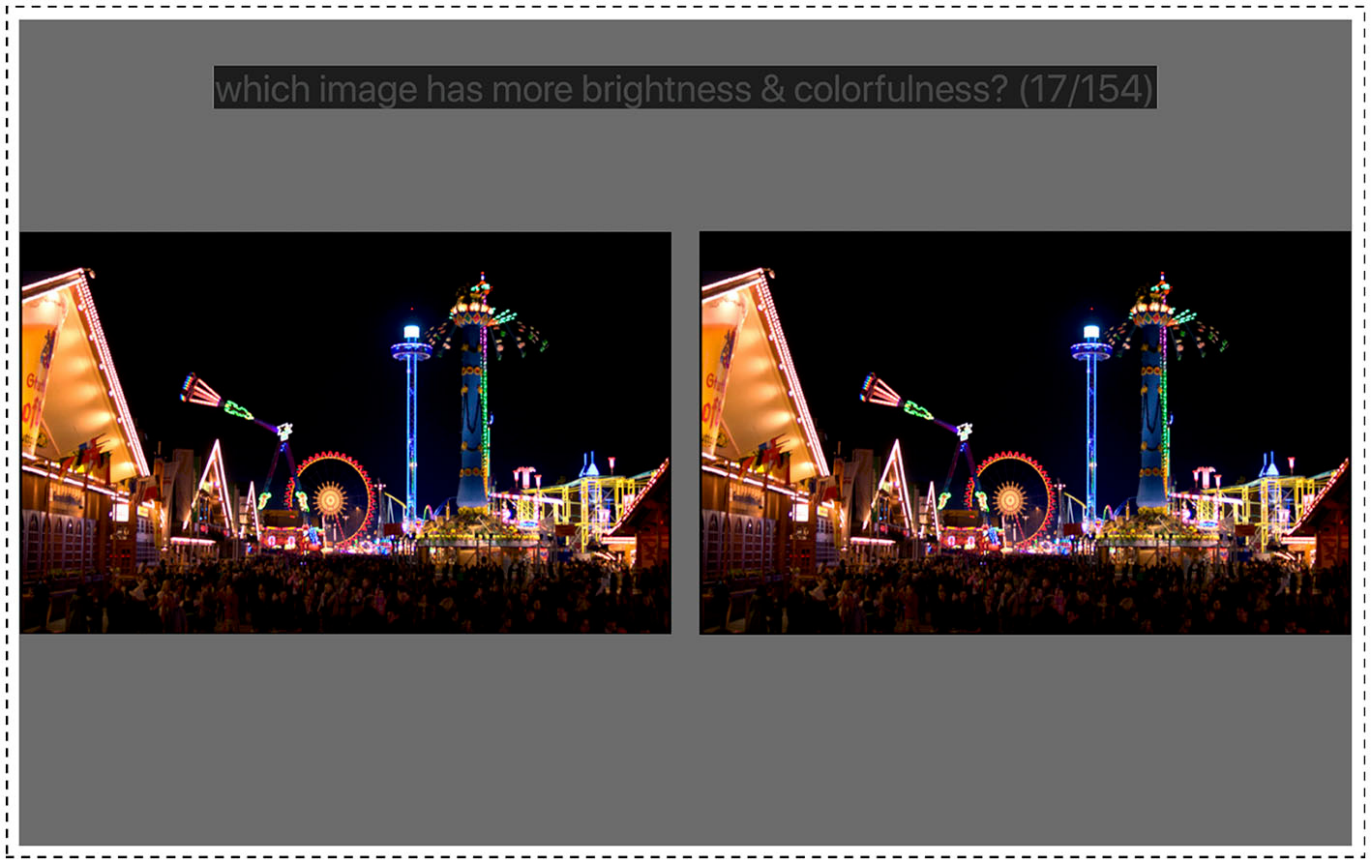
Stimulus configuration where the observers saw two images side-by-side and was asked to select the one with higher brightness and colorfulness. The background is a uniform gray at 100 nits surrounded by a thin white border of 500 nits. Source: image stimuli from the dataset in (Froehlich et al., 2014).

To evaluate the perceptual impact of different gamut settings, a psychophysical experiment using paired comparisons between those gamuts was conducted. The experiment was done on an Apple Pro XDR display, which could render a peak luminance of 1,500 nits with P3 RGB primaries. Figure 2 shows the stimulus configuration, where the whole screen covered a field of view (FOV) of 39 × 22 visual degrees at ~1 m viewing distance. Two images with the same content but different gamut parameters were shown side by side with a one-degree separation. Each image covered an FOV of 20 × 11°. The background was a uniform gray at 100 nits surrounded by thin white borders (500 nits, 0.5°), which together serve as the adaptation background. The white point at 500 nits D65 was also used in the following CGV calculations for conversion into CIELAB. The image was surrounded by thin black borders. On the top of the screen, there was an instruction text box which is further described in the following section.

**Figure 3:**
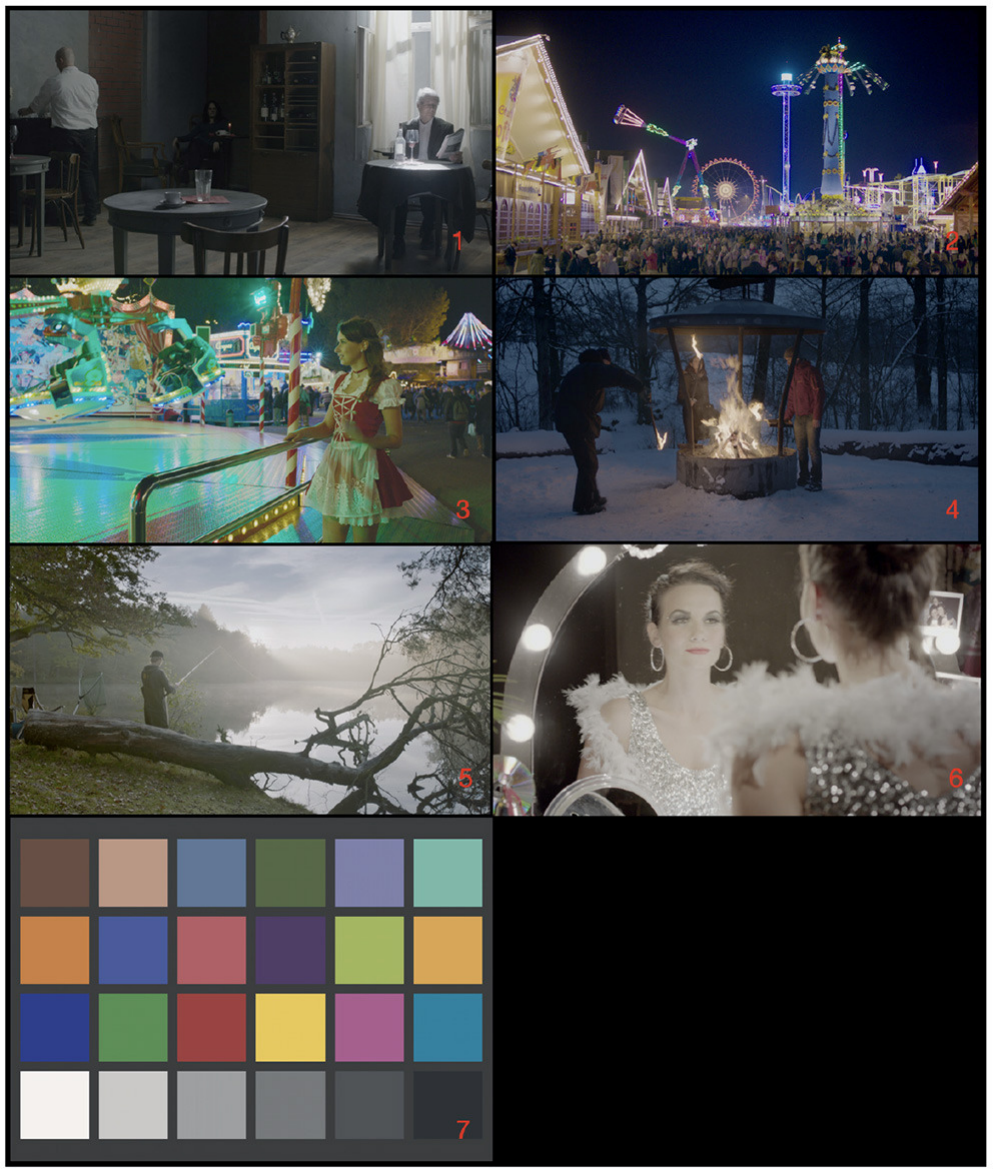
Image contents including six images selected from the HdM dataset (Froehlich et al., 2014) and one color-checker. The image index is labeled in the bottom-right corner.

The images used in the experiment are presented in Figure 3. The original images were selected from the HdM HDR dataset (Froehlich et al., 2014) which was curated for cinematic contents covering different semantic scenes/objects and different dynamic ranges. The subset of six images we selected was also intended to cover its representatives in these two aspects. In addition, a Macbeth color-checker uniformly lit with a 1,400 nits CIE D65 was supplemented. Color-checker has no meaningful object identities; thus it might help separate the contribution of attention to any specific image regions, and as will be shown in the result section, its uniform color patches are easier to compare and might more simply correspond to what a wider gamut entails. The seven images were modified according to the gamut and color conversion settings described above, and their colorimetric values were converted to the RGB encoding of the Apple XDR. As we linearly scaled the peak luminance from 4,000 nits (the original dataset) to 1,500 nits (the XDR peak), the actual appearance to the observers might not match the visualizations in Figure 3, especially the dark regions were more likely to be compressed thus lose details. However, these operations are expected to have little impact on the image attributes of our interest, i.e., brightness and colorfulness. Although the colorimetric values of images might be impacted by some spatial processing or artifacts native to the display, we believe the symmetric configuration and left-right order randomization would balance the potential impact.

Our experimental protocol has received IRB approval from our institution. Thirty-three observers, aging from 17 to 55 (mean of ~23), participated in the experiment. Most of them were college students, and approximately one-third had a color science background. All the observers had a normal color vision as verified by the Ishihara test. After the observers entered the room, they signed the consent form, adjusted the chair such that their eye level aligned with the center of the screen, and went through a 2 min dark adaptation before the experiment.

The task was to choose from a pair of images which one had higher brightness and colorfulness, which are considered to be the most relevant image quality attributes for the upper boundary of the gamut we would like to probe. Gamut and its utilization can affect image attributes in different dimensions. Previous work from Jiang et al. (2020) asked observers to select “which image is more colorful and detailed.” Since we considered that detail level might be more related to quantization level (color depth) and how colorists take advantage of the gamut, brightness and colorfulness are preferred to gauge the upper boundary of a gamut. The color shifts between the displays simulated in this paper happened along both lightness and chroma axes in a correlated way (which can be observed from the symmetric relation shown in **Figure 6**). The perceived gamut volume is thus expected to receive joint contributions from the two dimensions, corresponding to perceived brightness and colorfulness, respectively. Therefore, it suffices to combine the two perceptual attributes to be judged together. Another difference in our experimental design from Jiang et al. (2020) is that we adopted a side-by-side comparison instead of a temporally successive comparison. Their interest was the peak luminance level (adaptation independence), whereas our objective assumed a constant adaptation level. During the pilot experiment, it was established that the temporal comparison scheme was more difficult for the observers to detect the difference, even with a shorter time interval. Our observers did not have any time constraints in their decision-making, and there was a 1 s interval between trials with only the background and no images.

For each image content, paired comparisons between 5 pairs of gamut parameters led to 10 trials. And for each compared pair, the left-right order was repeated for both conditions. In addition to those 140 trials (10 comparisons across 7 images with 2 spatial arrangements of the images), we added a set of 14 trials where the #1 gamut and its 30% luminance off version (the image luminance was adjusted with a scalar of 0.7), each image left-right switch repeated, were also added to as a sanity check and also an indicator of the task difficulty. On average, it took each observer 30 min to complete the 154 trials in random order.

## 3. Results

### 3.1. Observer Screening and Image CGVs

**Figure 4:**
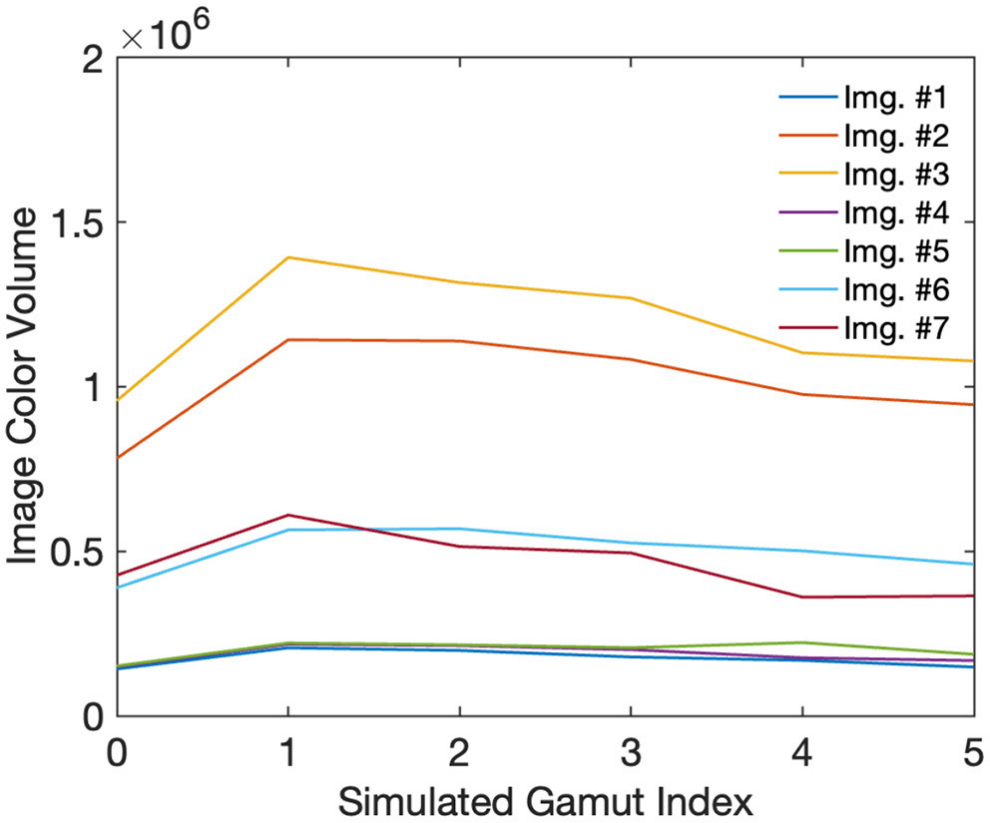
Image CGVs across different gamut conditions. Each line corresponds to one image content. Gamut #0 was the checking condition, which was 30% luminance off from Gamut #1. The same image content with a higher CGV was expected to have higher brightness and colorfulness.

By screening the correction rate of the 14 checking trials, only 11 observers out of all 33 observers achieved a 100% correct selection rate, with the lowest result being 10 correct answers out of 14. Figure 4 shows the CGVs calculated for each image with different gamuts. In particular, Gamut #0 is the checking condition, which is the same as Gamut #1 at 30% lower luminance. Interestingly, 30% reduction in luminance led to 30% reduction in CIELAB volume after those non-linear transformations. However, the good-observer ratio of 11/33 implies the difficulty of seeing the perceptual differences when there was a global level of luminance changes. Also of note is that across the five gamuts, the lines for each image approximately, but not exactly, follow the trend of the CGVs in Table 1. More quantitatively, the average image-wise CGVs are listed in Table 2. Thus, a larger boundary CGV generally correlates with larger image CGVs, but the exception cases also mean there is some impact from the parameters that are relevant to the gamut interiors, in our case, due to different white channel activation levels. In particular, images #2, 3, &7, whose original CGV for gamut #1 was relatively high, had more variations in CGV across different gamuts. Those images either had more colorful neon lights or, for the colorchecker case, were closer to the upper gamut boundary. In contrast, the other images had more similar hues. Thus, their pixels tended to be located in the interior and lower region of the original source gamut, which were more likely to be invariant to the parameter changes across the five simulated gamuts.

**Table 2:** CGV for both gamut boundary and all images averaged.

**Simulated gamut**	**#1**	**#2**	**#3**	**#4**	**#5**
CGV (*10^6^)	3.72	1.23	1.76	0.75	1.01
Mean image-wise CGV (*10^6^)	0.62	0.59	0.57	0.50	0.48

*The boundary CGVs are those in Table 1*.

### 3.2. Perceived CGV Scales

**Figure 5:**
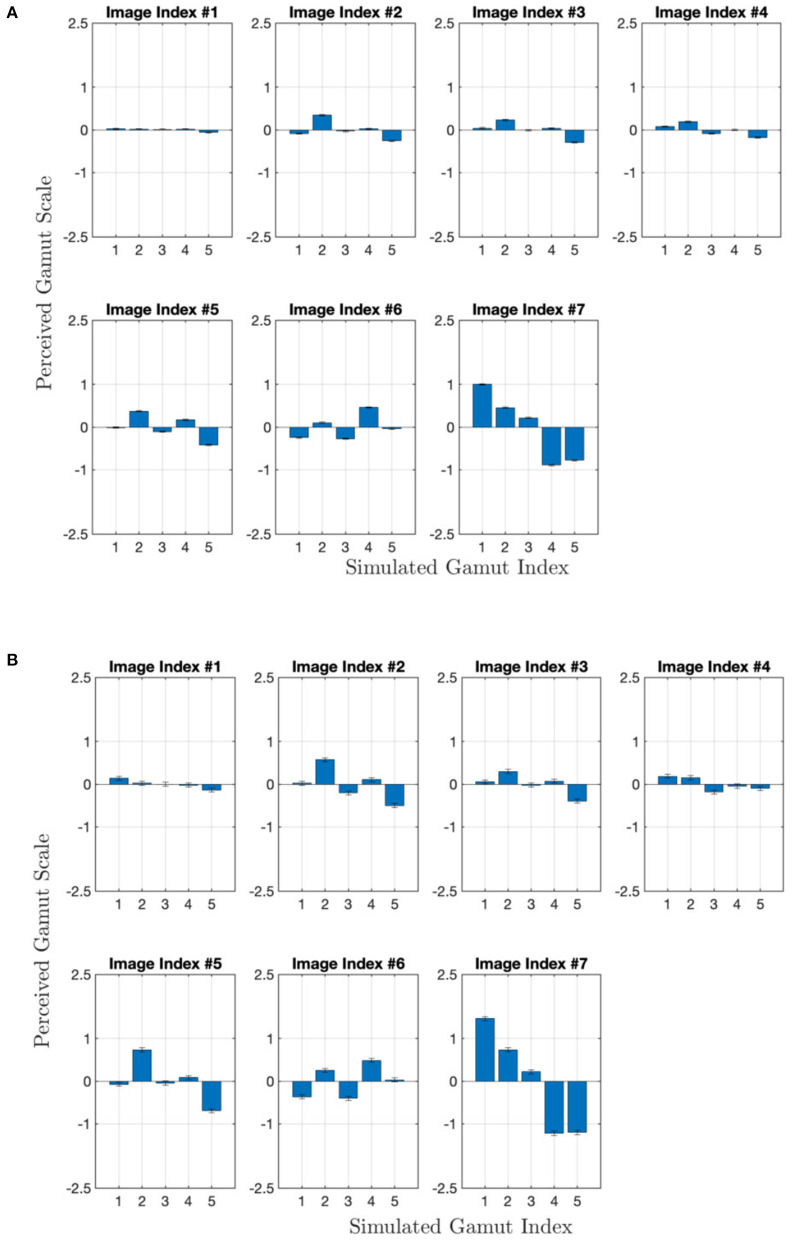
Perceived CGV scales, derived from all 33 observers **(A)** the subset of 11 observers **(B)**, vs. different gamuts. Each panel corresponds to one image content's results.

Based on the assumption of Thurstone Case V, the comparison voting results of those 140 trials were converted into perceived gamut scales. The scales as z-scores have a unit of one standard deviation from the standard normal distribution, corresponding to the probabilities of 0.159 (z-score of −1) or 0.841 (z-score of 1). Here higher values mean larger perceived CGV or brightness and colorfulness specifically. Both results from all observers and the 11 “good” observers are shown in Figure 5. The error bars were calculated as a function of the number of gamuts (5) and the number of observers times their repetitions (Montag, 2006). They mostly align with each other, and the good-observer results show more discrimination distances, e.g., Image Index #1 & 7, which follow what the image-wise calculated CGVs predicted. For other image contents, one observation is that Gamut #2 has higher perceived scales than Gamut #5. The two gamuts have different WLO/CLO's, and the results follow what calculated CGVs predict too. Regarding other gamut comparisons, we instead investigated what happened with the manipulations of the color conversion algorithm did by comparing the color differences between image pairs, which is more directly connected to how observers reached their selection decision in each trial. In the following, the 11-observer results are primarily used.

### 3.3. Image Color Difference Analysis

**Figure 6:**
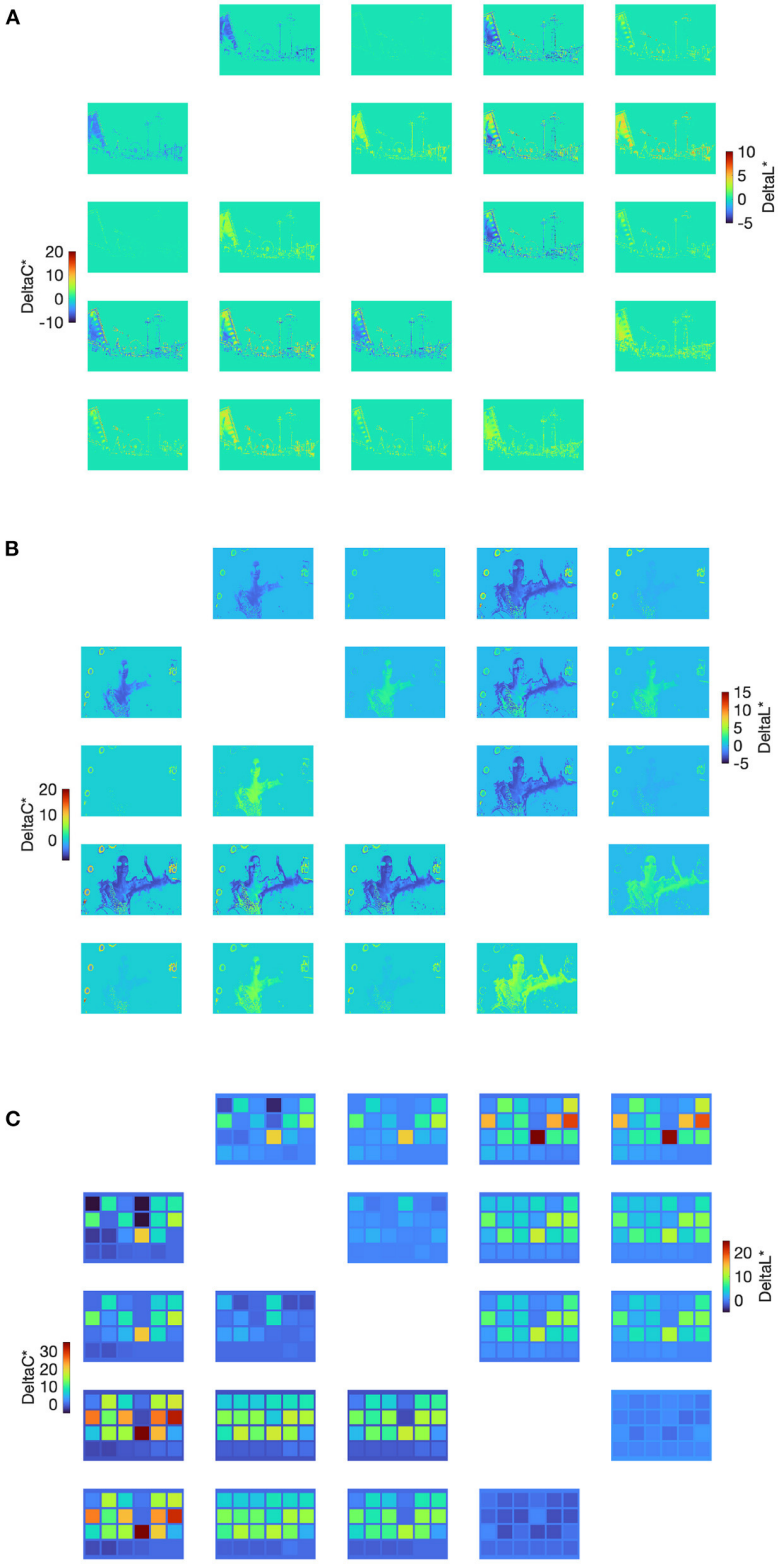
Image color differences for both CIELAB lightness and chroma between different pairs of gamuts for image #2 **(A)**, image #6 **(B)**, and image #7 **(C)**. The 5 × 5 image matrix (the diagonal skipped) include both lightness difference (the upper-right triangle) and chroma difference (the lower-left triangle) that are diagonally symmetric.

An image-wise analysis can be useful to decouple the compound effects between display color gamut and image content. And if image-wise analysis can provide reasonable explanations for a group of representative image contents covering the whole gamut, it would be useful to generalize to predict any images (and hopefully the average results across all images would converge to the display color gamut). As the task involved comparing brightness and colorfulness and the color conversion algorithm considered minimal hue shifts, we calculated the image color difference using lightness and chroma dimensions in CIELAB, shown in Figure 6 below. In each sub-figure, the 5 × 5 image matrix (the diagonal skipped) includes both lightness difference (the upper-right triangle) and chroma difference (the lower-left triangle), represented as heat-map style images where the pseudo-colors correspond to the difference. For each difference image at (i, j) for lightness and (j, i) for chroma, where i and j correspond to two gamut indices, the difference was always calculated as (i–j), thus the matrix is approximately mirror-symmetric as lightness and chroma correlate (which was prioritized in the color conversion algorithm).

If the image difference ideally follows the image-wise CGV trends across the five gamuts, the color difference would be mostly positive between (i–j) when i is smaller than j, and the larger interval between i and j, the larger the color difference. This is observed for the color-checker case (Figure 6C), which combined with the largest color difference magnitudes explains why the color-checker result followed the prediction above. However, the position where the white channel starts to activate apparently affects the gamut interiors. For example, Gamut #1 vs. #2 and #1/2/3 vs. #4 lead to both positive and negative color differences for those pairs, which may make those comparison trials ambiguous. Despite this, in most cases, the perceived scales between a pair of gamut conditions can be related to their corresponding image color differences.

## 4. Discussion

In this work, the perceived color gamut of images was studied from both computational and psychophysical perspectives. As the determination of display gamut boundary ignores the image content dependency, an image-wise analysis including both color gamut volume per image and color difference across images was conducted. The psychophysical results can be well predicted and explained by such image-wise analysis which captures how colors both on the gamut boundary and in the interior are manipulated according to different gamut conditions, as well as the spatial distribution of gamut mapping errors. As our selected stimuli covered a group of representative image contents from the whole gamut, it would be promising to generalize to predict any image, with a colorimetrically explicit forward model, and hopefully, the average results across all images would better reflect the usefulness of a given display gamut design. A similar framework has also been recently proposed (Lee et al., 2020). While the simplistic and colorimetric comparison between two images has been shown to be predictive, future work can explore image color appearance modeling (Johnson et al., 2010) and spatial gamut mapping (Bonnier et al., 2006; Vazquez-Corral and Bertalmío, 2018), especially when there are more variations in display parameters and image contents.

However, because this study considered only one display gamut with no white channel contribution, these results apply specifically to color gamut volume comparisons between RGBW and RGB-only displays. As such, they cannot be extended to comparison within the RGB-only class of displays, where the color gamut volume calculation has proved to correlate well with performance. Also, for the color conversion and gamut mapping component, when considering other advanced options (Morovič, 2008; Zamir et al., 2019), their impact on brightness and colorfulness attributes needs further investigation.

The CGV was primarily calculated in the CIELAB space. Although a Euclidean color space that is uniform across the whole gamut might be hard to develop, a more uniform color space is potentially more meaningful for calculating the overall CGV size. Previous results between CIELAB and CIECAM02 had not found a significant difference (Jiang et al., 2020). Dimension independence, i.e., a lack of correlation between individual chroma and luma dimensions, is useful for determining the impact in each dimension. The CIELAB space does not perform well in this regard because the lightness L* dimension only considers partially perceptual lightness without incorporating higher-order effects such as the Helmholtz–Kohlrausch effect. Park and Murdoch (2020) studied the trade-off between chromaticity gamut area and luminance for image quality and found more chroma can equivalently compensate for the deficiency in lightness. Recent efforts have been made to address this problem by adding the compensation component in gamut mapping (Zamir et al., 2019) and proposing more independent color scales (Xie and Fairchild, 2021). Our future work includes CGV re-calculations in such a new space.

## 5. Conclusion

Using RGBW displays (RGB plus a white channel) as a special case, the results of the psychophysical study demonstrated that a larger display gamut enclosed by the boundary does not guarantee a larger color gamut perceived in images. Five gamuts with different white channel contributions were simulated, and seven different image contents were curated and rendered on each display. Using a paired comparison experiment with 33 observers, the perceived scales of color gamut as perceived brightness and colorfulness were derived. The results show more correlations with the image-wise than display-wise CGV and can be explained with image color differences. Our findings highlight the importance of considering image contents when optimizing display gamut volume, which can be guided by such image-wise analysis.

## Data Availability Statement

The original contributions presented in the study are included in the article/supplementary material, further inquiries can be directed to the corresponding author/s.

## Ethics Statement

The studies involving human participants were reviewed and approved by Heather Foti, Institutional Review Board for the Protection of Human Subjects in Research, Rochester Institute of Technology. The patients/participants provided their written informed consent to participate in this study.

## Author Contributions

HX performed the experiment conduction and result analysis, and wrote the first draft of the manuscript. All authors contributed to the result discussion and article revision and approved the submitted version.

## Funding

This work was sponsored by LG Electronics.

## Conflict of Interest

RW was employed by company LG Electronics/Zenith R&D. The remaining authors declare that the research was conducted in the absence of any commercial or financial relationships that could be construed as a potential conflict of interest. The authors declare that this study received funding from LG Electronics. The funder had the following involvement in the study: providing the source code for the color conversion algorithm and approving the decision to publish this article.

## Publisher's Note

All claims expressed in this article are solely those of the authors and do not necessarily represent those of their affiliated organizations, or those of the publisher, the editors and the reviewers. Any product that may be evaluated in this article, or claim that may be made by its manufacturer, is not guaranteed or endorsed by the publisher.
